# SapBark-64: A dataset of bark images for 64 fruit-tree sapling classes

**DOI:** 10.1016/j.dib.2025.112354

**Published:** 2025-12-03

**Authors:** Sayyad Alizadeh, Hamed Shamsi

**Affiliations:** a Faculty of Science, Department of Software Development, Karadeniz Technical University, 61080, Trabzon, Turkey; b Faculty of Engineering, Department of Metallurgical and Materials Engineering, Karadeniz Technical University, 61080, Trabzon, Turkey

**Keywords:** Tree bark images, Tree identification, Bark texture analysis, Sapling image, Deep learning, Tree dataset, Horticulture

## Abstract

We present SapBark-64, a curated dataset of 5742 close-range bark images from 64 fruit-tree sapling classes (species/cultivar). Images were acquired in situ at three commercial nurseries in Trabzon (Türkiye) in 2025, targeting 1–2-year saplings routinely traded in nurseries. Photographs were captured with an iPhone 16 Pro Max at approximately 10 cm from the trunk under near-uniform illumination, using a white background to occlude scene clutter and preserve fine-scale morphology. For each class, a nursery label photo was recorded to support ground truth, and class-level characteristics were collected at the time of recording under expert supervision.

The repository is organized as two parallel image folders plus a structured metadata workbook: (i) raw images (JPG) and (ii) background-removed images (WebP) that mirror the same 64 class folders named by species/cultivar, enabling one-to-one pairing across versions; and (iii) an Excel (XLSX) metadata file list- ing standardized fields (family, scientific/common name, cultivar/variety, sapling height, trunk diameter, best planting season, growth rate, fruit-bearing age, average yield, production region, propagation method). This organization facilitates fine- grained identification and retrieval tasks and supports trait-conditioned analyses linking visual texture to horticultural attributes.

The dataset is publicly available in an open repository under a permissive license; acquisition conditions, directory layout, and the metadata schema are documented to enable unambiguous reuse.

Specifications Table

**Table:** 

Subject	Computer Vision; Agricultural Informatics; Plant Phenotyping; Decision Sciences
Specific subject area	Identification of *species*/cultivars from bark images of fruit-tree saplings (1–2 years); texture-based classification and image retrieval
Type of data	Images; Tables
Name of dataset	SapBark-64
How the data were acquired	Images were captured in situ with the rear camera of an iPhone 16 Pro Max at three commercial nurseries in Trabzon (Türkiye) during late April–mid May 2025. Bark was photographed at approximately 10 cm standoff with a white background under near-uniform illumination.
Parameters for data collection	Commercial 1–2-year-old saplings; imaging of the trunk/bark region; multi- ple images per class subject, depending on stock availability.
Description of data collection	Accessible specimens from each class were photographed, yielding 57– 149 images per class and a total of 5742 images across 64 classes. The photography process was supervised by an agricultural expert, and class- level metadata were recorded at capture time. Saplings were classified at the species–cultivar level; for example, for *Ficus carica*, the Bardacık, Patlıcan, and Black cultivars are included.
Data format	Raw images: JPEG; Background-removed images: WebP; Metadata: XLSX
Data source location	City/Province: Trabzon; Region: East Black Sea; Country: Türkiye (saplings supplied from Aegean, Marmara, Mediterranean, and Central Anatolia regions).
Measurement units	Height: cm; Trunk diameter: mm/cm; First fruiting age: years; Yield: kg
Raw or processed	Raw field images are provided; for bark-focused operations, derived versions with background removed are provided in a parallel directory.
Data accessibility	Repository: Zenodo; doi:10.5281/zenodo.17196298;
Ethics statement	No human or animal data; imaging conducted in commercial areas with permission.

## 1 Value of the Data

• Closes a key gap for early-stage saplings. Public dataset of 5742 close-range bark images from 64 fruit-tree sapling classes (1–2 year, species/cultivar level), enabling fine-grained benchmarks specific to nursery practice.
• Direct practical use in nurseries. Supports label verification, inventory quality control, and farmer training; images and schema are suitable for mobile/edge deployment of deep-learning models.
• Ready for computer-vision pipelines. Two parallel image sets, raw and background-removed allow controlled comparisons for classification and retrieval without extra preprocessing.
• Rich metadata for trait-aware analysis. Harmonized class-level fields (family, scientific/common name, cultivar, height, trunk diameter, planting season, growth rate, fruit-bearing age, average yield, region, propagation) enable trait-conditioned error analysis and cross-family generalization.
• Testbed for few-shot and robustness. Subtle inter-cultivar texture differences suit few-shot learning, domain adaptation, and open-set/robustness studies.

## 2 Background

Accurate identification of fruit tree saplings at the cultivar level is of practical importance. It is essential for nurseries and growers because it underpins quality assurance. It also helps in disease control. It enables traceability. And it supports planting decisions. Young saplings (1–2 years old) are difficult to identify in practice because their physical characteristics are not yet fully developed. Many saplings also look very similar at this stage. In greenhouses, incorrect labeling is usually not apparent until fruiting (3–5 years). This can create an avoidable economic risk. As production and retailing frequently occur in different regions, and on-site taxonomic expertise at the point of sale can be limited, buyers often rely solely on the seller’s label. Conventional recognition cues based on leaves, flowers, or fruit are season-dependent and sensitive to phenology and weather. By contrast, bark texture is observable year-round and relatively stable, making it a suitable biological target for automated recognition in nurseries and supply chains.

To fill this gap, we compiled the SapBark-64 dataset. This dataset contains 5742 close-up images of the bark of fruit tree saplings. The images correspond to 64 classes (cultivars) of saplings. The data were collected under controlled conditions in three nurseries in Trabzon (Turkey, 2025). Each class is accompanied by consistent metadata. This metadata includes family, scientific/common name, cultivar/variety, sapling height, trunk diameter, best planting season, growth rate, fruit-bearing age, average yield, production region and propagation method. The dataset provides a consistent basis for objective, fine-grained evaluation of species/cultivar recognition and related bark-based analyses in nursery practice.

Prior work demonstrates that image-based plant and tree-bark datasets are effectively used with deep learning for fine-grained identification. CNN-based studies on mature-tree bark (e.g., BarkNet 1.0 and follow-ups) report high accuracies with ResNet/EfficientNet variants and visual explanations (CAM) [[1], [2], [3], [4], [5]]. Complementary handcrafted-descriptor baselines (e.g., ILTP, multi-scale LBP + SVM, SMBP) further validate the utility of bark texture as a discriminative cue [[6], [7], [8], [9]]. Recent datasets such as BarkVN-50 and CentralBark expanded class diversity and confirmed scalability of CNNs to large bark corpora [3,10]. Closer to our scope, the AlmBark dataset showed that cultivar-level discrimination is also feasible on young almond bark using hybrid wavelet + CNN pipelines [11]. These studies collectively justify that bark-image datasets can be directly leveraged by deep models for fine-grained recognition, motivating the release of SapBark-64 for early-stage saplings.

## 3 Data Description

**Fig. 1:**
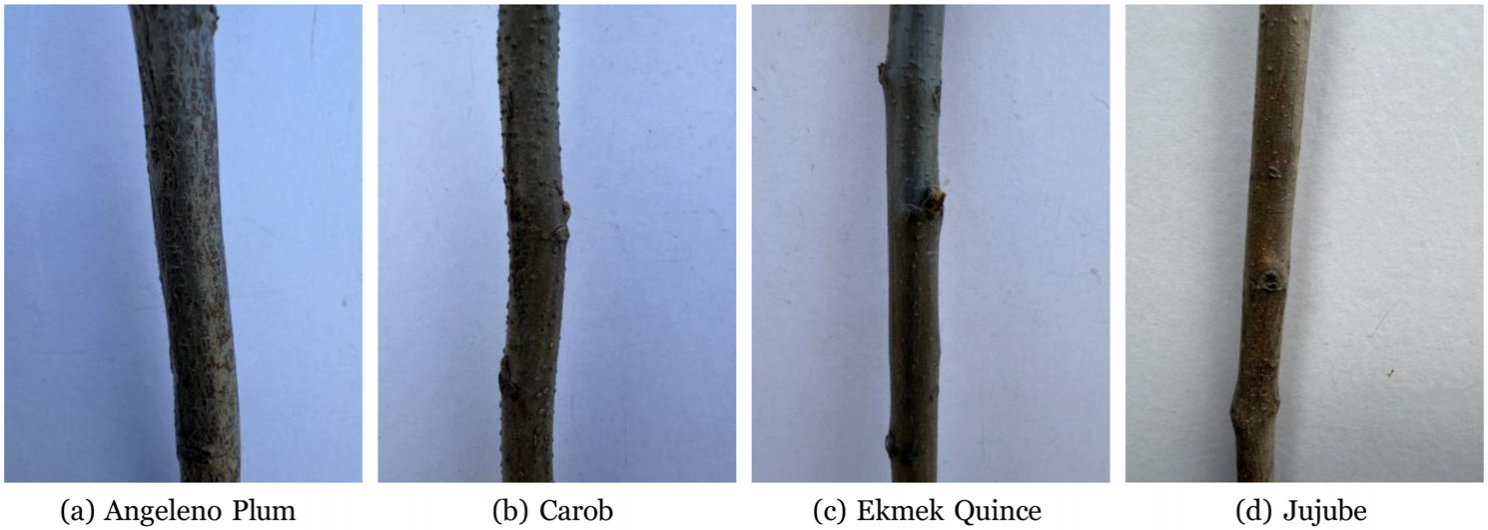
Four raw image samples.

The SapBark-64 release comprises high-quality, close-range photographs of the bark of 64 fruit-tree sapling classes (species/cultivar). In total, 5742 bark images were collected in nurseries in Trabzon (Türkiye) under expert supervision, with per-class counts ranging from 57 to 149 depending on stock availability. All photographs were taken at approximately 10 cm standoff from the trunk using an iPhone 16 Pro Max to preserve fine-scale morphological texture; a white background was used to occlude scene clutter and illumination was kept as uniform as practical. Fig. 1 illustrates representative samples from different classes. For each class, a sticker photo of the nursery sticker has been added; these 64 label images are in addition to the 5742 images of saplings. Raw RGB images were saved as JPG files at 3024 × 4032 pixels.

The deposit dataset SapBark-64 contains two parallel image directories and one structured metadata file. The raw images directory includes 64 class folders (one per species/cultivar), named with the class identifier as used on the nursery labels; each folder holds the bark photographs for that class. The background- removed images directory mirrors the same 64 class folders, providing per-image counterparts that suppress the background while preserving the bark region. In addition, a single Excel (XLSX) metadata workbook accompanies the images. Table 1 shows the frequency of images for a limited number of species, along with their common names.

**Table 1:** Species, cultivar, common name, and number of images of each class.

*Species*	Cultivar	Common Name	Images
*Crataegus azarolus*	Red Hawthorn	Red Azarol	83
*Cydonia oblonga*	Ekmek	Ekmek Quince	87
	Eşme	Eşme Quince	91
	Bilecik	Bilecik Walnut	92
*Juglans regia*	Şebin	Şebin Walnut	87
	wild	Wild Walnut	90
	Angeleno	Angeleno Plum	82
	Black Diamond	Black Diamond Plum	85
*Prunus salicina*	Fortune	Fortune Plum	76
	Show Time	Show Time Plum	84
	Black Splendor	Black Splendor Plum	86
*Corylus avellana*	general type	Hazelnut	105
	Bardacık	Bardacık Fig	146
*Ficus carica*	Patlıcan	Patlıcan Fig	80
	Black	Black Fig	149
*Ceratonia siliqua*	general type	Carob	75
*Actinidia deliciosa*	Hayward	Kiwi	85
*Punica granatum*	Devedişi	Pomegranate	67
	White	White Nectarine	80
*Prunus persica* var. *nucipersica*			
	Red	Red Nectarine	76
*Citrus sinensis*	general type	Orange	88
	Hale	Hale Peach	79
*Prunus persica*	Redhaven	Red Haven Peach	93
	Yellow	Yellow Peach	78
			

**Fig. 2:**
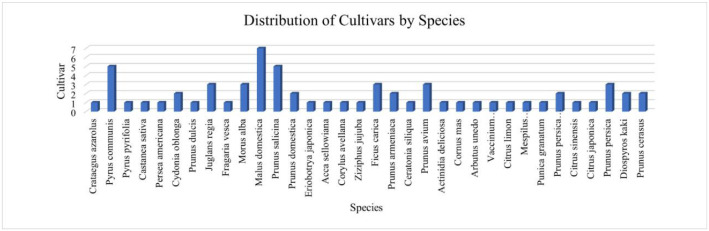
Distribution of cultivars by species.

To summarize the taxonomic structure, a table and a bar chart report the number of cultivars per species Table 2 and Fig. 2. In this dataset, the distribution is heterogeneous; for example, Malus domestica includes 7 cultivars, Prunus salicina 5 cultivars, Pyrus communis 5 cultivars; several taxa have 3 cultivars (e.g., Ficus carica, Morus alba, Juglans regia, Prunus avium, Prunus persica), while others have by 1–2 cultivars. In this case, there are a total of 34 species and 64 cultivars of sapling bark images in the SapBark-64 dataset.

**Table 2:** Distribution of cultivars by species.

No.	Species	Cultivar
1	Crataegus azarolus	1
2	Pyrus communis	5
3	Pyrus pyrifolia	1
4	Castanea sativa	1
5	Persea americana	1
6	Cydonia oblonga	2
7	Prunus dulcis	1
8	Juglans regia	3
9	Fragaria vesca	1
10	Morus alba	3
11	Malus domestica	7
12	Prunus salicina	5
13	Prunus domestica	2
14	Eriobotrya japonica	1
15	Acca sellowiana	1
16	Corylus avellana	1
17	Ziziphus jujuba	1
18	Ficus carica	3
19	Prunus armeniaca	2
20	Ceratonia siliqua	1
21	Prunus avium	3
22	Actinidia deliciosa	1
23	Cornus mas	1
24	Arbutus unedo	1
25	Vaccinium corymbosum	1
26	Citrus limon	1
27	Mespilus germanica	1
28	Punica granatum	1
29	Prunus persica var. nucipersica	2
30	Citrus sinensis	1
31	Citrus japonica	1
32	Prunus persica	3
33	Diospyros kaki	2
34	Prunus cerasus	2
	Total Cultivar/Class	64

The Excel file provides class-level attributes recorded at capture time. It lists the general attributes for each class, including Common Name, Scientific Name, Fruit Tree Type, Plant Family, Cultivar/Variety, Sapling Height, Trunk Diameter, Best Planting Season, Growth Rate, Fruit-bearing Age, Average Yield per Mature Tree, Type of Fruit, Region where the sapling is grown, Propagation Method, and the per-class image count. Table 3 shows a summarized example of a few selected fields of class-level metadata for a sapling species.

**Table 3:** Sample of a few class-level metadata fields.

General Characteristics	Value
Scientific Name	Crataegus azarolus
Sapling Height	40 cm – 1.5 m
Trunk Diameter	0.5 – 2 cm
Fruit-bearing Age	4 – 6 years
Average Yield per Mature Tree	10 – 30 kg
Number of images	83

## 4 Experimental Design, Materials, and Methods

Images were acquired in three commercial nurseries in Trabzon (Türkiye) during the 2025 nursery season to assemble a standardized bark dataset of 1–2 year fruit-tree saplings. Classes were defined at the cultivar level (species where no named cultivar exists), and only healthy, sale-ready saplings were photographed under consistent capture conditions. The release is organized as two parallel image folders (original; background- removed) plus a structured metadata workbook, enabling one-to-one pairing and reproducible reuse. Class labels (species/varieties) were performed under the supervision of a qualified horticultural expert at the data collection site. A concise summary of the acquisition settings follows.

Acquisition summary:

• Sites and period: three nurseries in Trabzon (Türkiye), late April to mid May 2025.
• Target: 1–2 year fruit-tree saplings; healthy, sale-ready specimens.
• Camera and settings: iPhone 16 Pro Max; native 3024 × 4032 px; 72 dpi; sRGB; handheld; no overlays.
• Capture geometry: 10 cm from trunk; white background to suppress clutter; near-uniform illumination.
• Per-class counts: 57–149 images depending on stock; total 5742 bark images; one label photo per class for ground truth (not counted).
• File formats: original JPG (3024 × 4032 px, 72 dpi, sRGB); background-removed WebP; class-level metadata in Metadata.xlsx (SI units).
• Organization: two parallel image folders (original, background-removed) with identical 64 class folders.
• Quality control: Visual checks were performed on all mis-segmented frames, corrected or removed.

### 4.1 Preprocessing

**Fig. 3:**
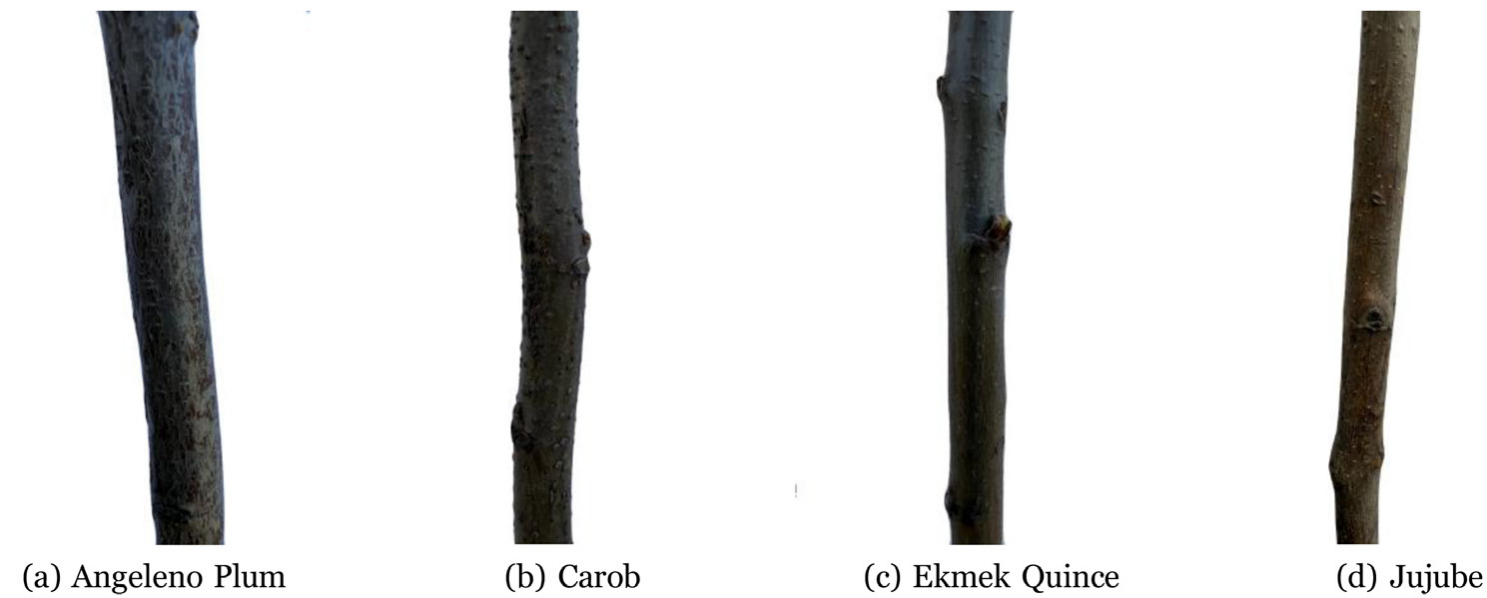
Four images with background removed.

Although images were acquired with a white occluder and near-uniform illumination, residual background content remained. To provide images that emphasize the sapling trunk, background removal was performed using a Fast Segment Anything (FastSAM)–based workflow [12]. A point near the trunk center was used to guide mask generation toward the bark region. Assuming the trunk occupies a smaller area than the background, a minimal-area mask selection heuristic was applied; if the smallest mask corresponded to background, automatic inversion corrected the assignment. The selected mask was then applied to the original image to generate a background-removed WebP file. Processing was executed batch-wise in Python while preserving folder names and class labels, so the background-removed directory mirrors the original hierarchy without relabeling. Finally, all background-removed images were manually checked, and none showed improper removal. Fig. 3 shows background-removed examples corresponding to the images in Fig. 1.

## Ethics Statements

No human participants or animal subjects were involved. The images depict cultivated fruit-tree saplings (1–2 years old) photographed in situ at commercial nurseries, with permission from nursery owners/vendors and under the supervision of an agricultural expert.

No identifiable persons, vehicle license plates, or proprietary documents were captured; the dataset contains no personally identifiable information. Geographic detail is limited to the city level (Trabzon, Türkiye), and business names/addresses are not disclosed.

Accordingly, institutional ethics approval and informed consent were not required under prevailing guidelines.

## Data Availability

All data are openly available on Zenodo at https://doi.org/10.5281/zenodo.17196298.

## Appendix Supplementary materials

Image, application 1
