# Supplementary figures and images for: SapBark-64: A dataset of bark images for 64 fruit-tree sapling classes
Source: Data Brief. 2025 Dec 3;64:112354. doi: 10.1016/j.dib.2025.112354 (PMC12756539; doi:10.1016/j.dib.2025.112354)

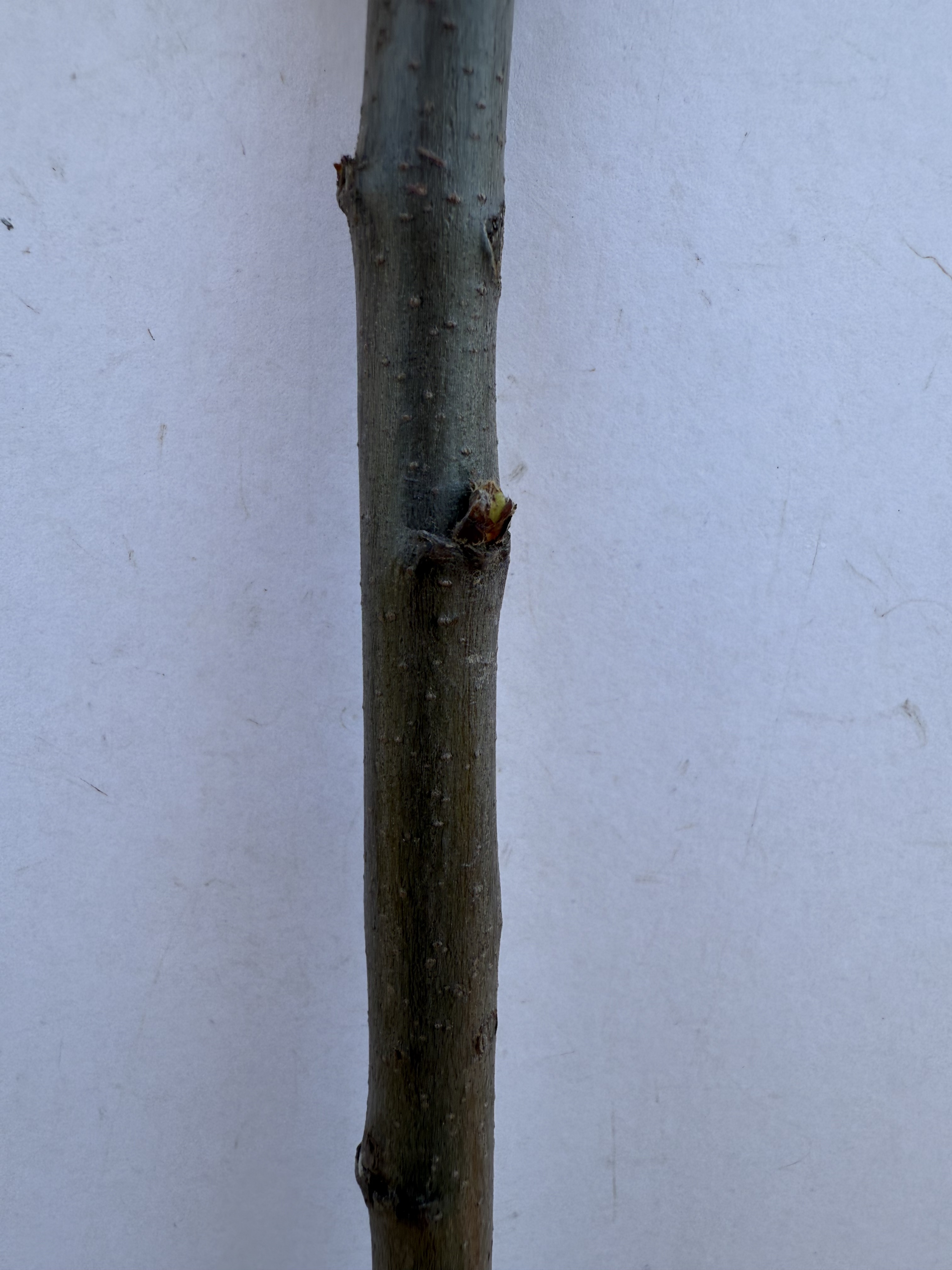

Supplement: Supplementary file 1 [file mmc1.zip › Ekmek Quince_Raw.JPG]

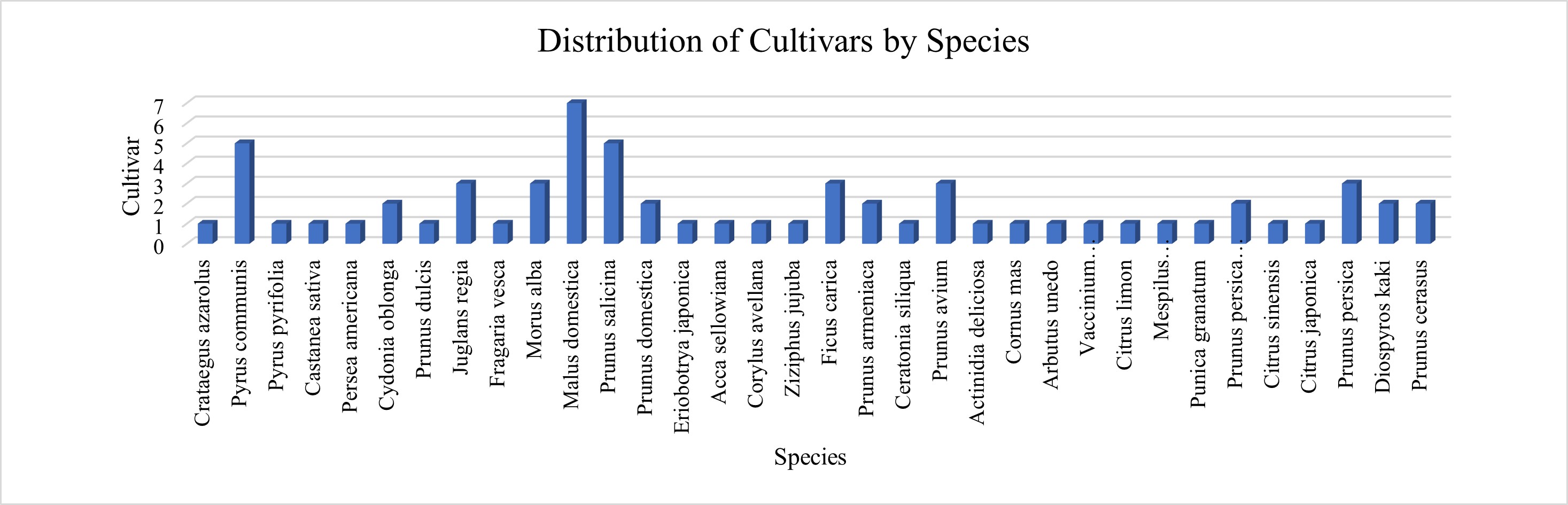

Supplement: Supplementary file 1 [file mmc1.zip › Distribution of Cultivars by Species.jpg]

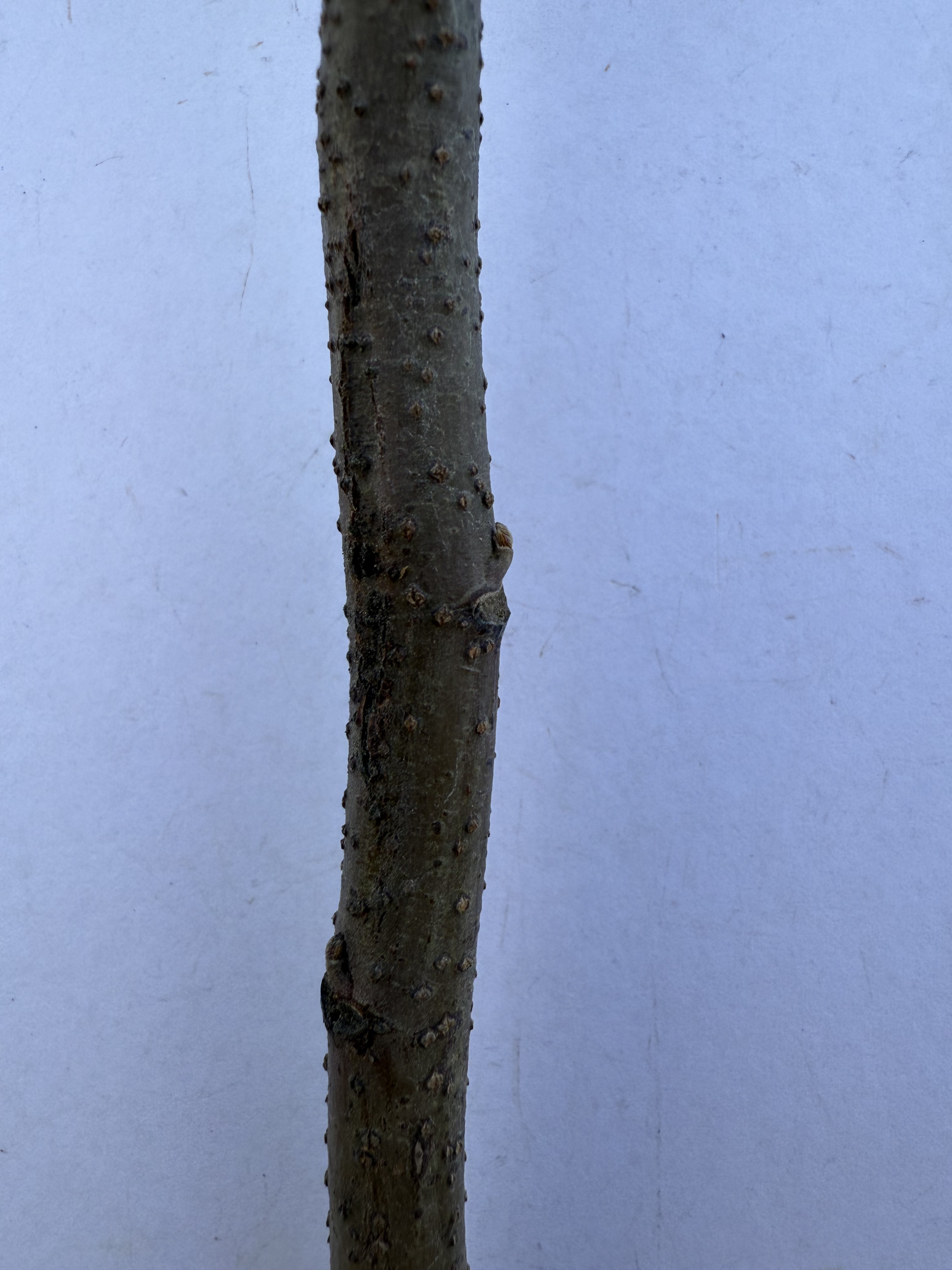

Supplement: Supplementary file 1 [file mmc1.zip › Carob_Raw.JPG]

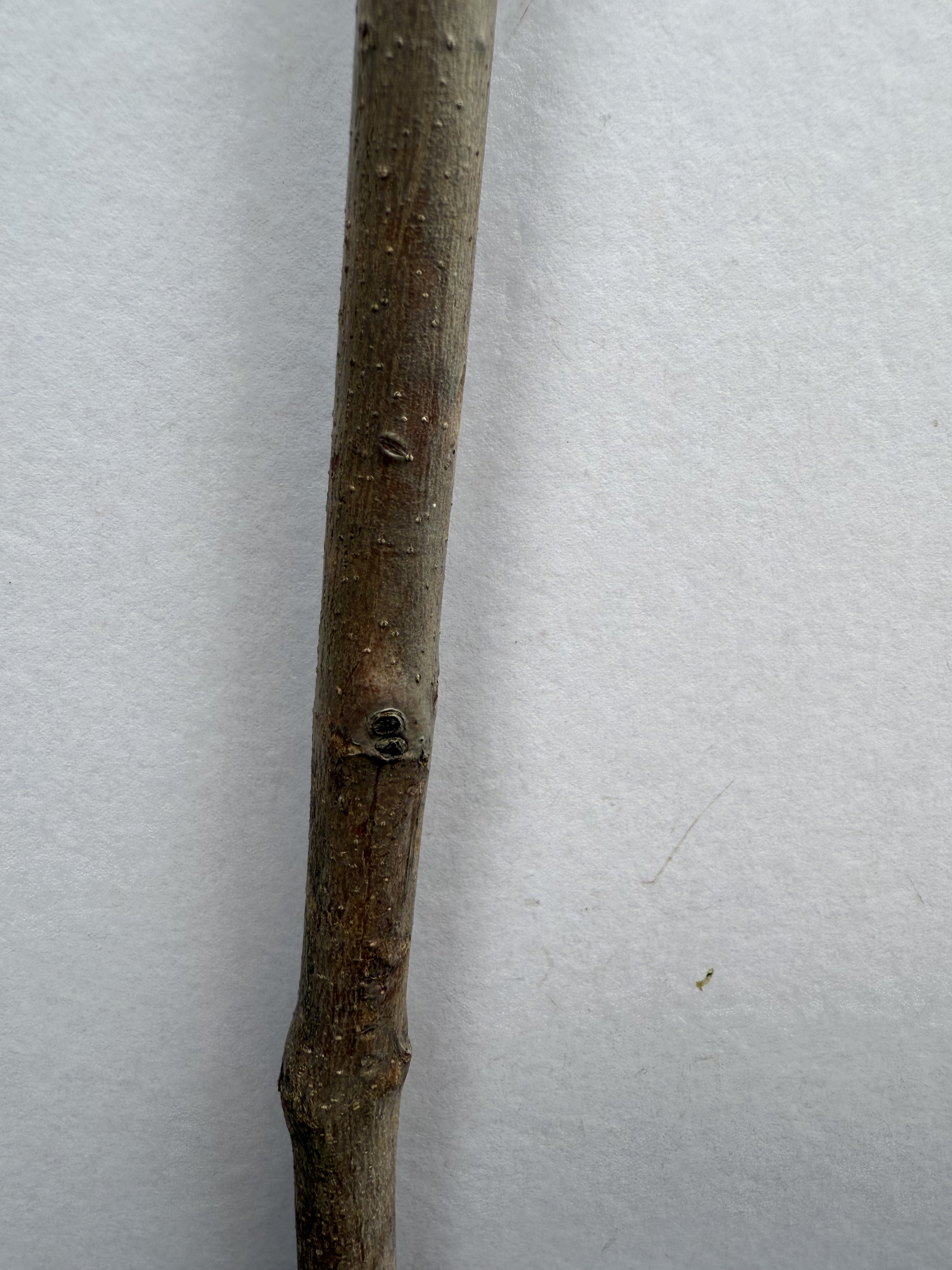

Supplement: Supplementary file 1 [file mmc1.zip › Jujube_Raw.JPG]

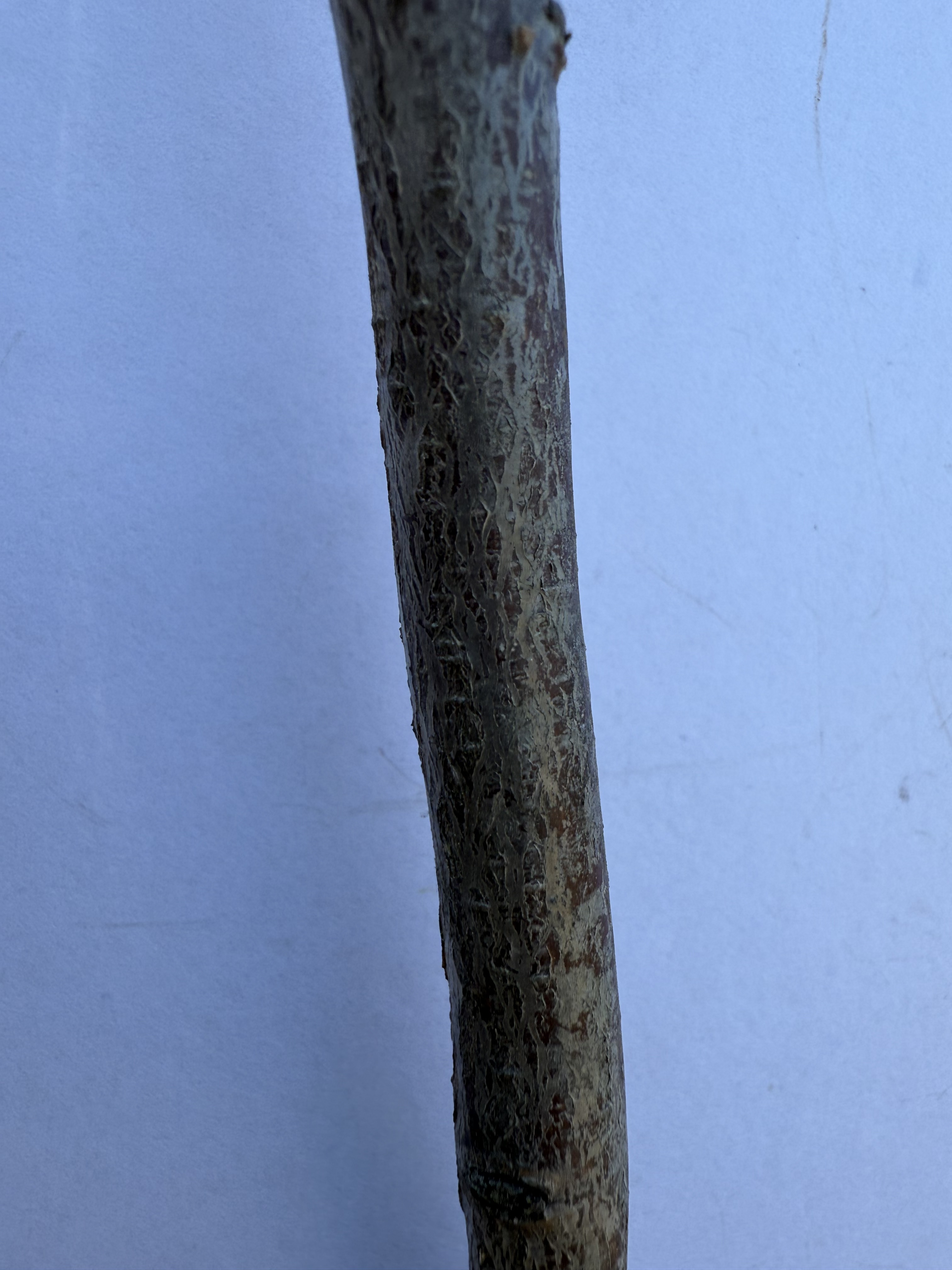

Supplement: Supplementary file 1 [file mmc1.zip › Angeleno Plum_Raw.JPG]

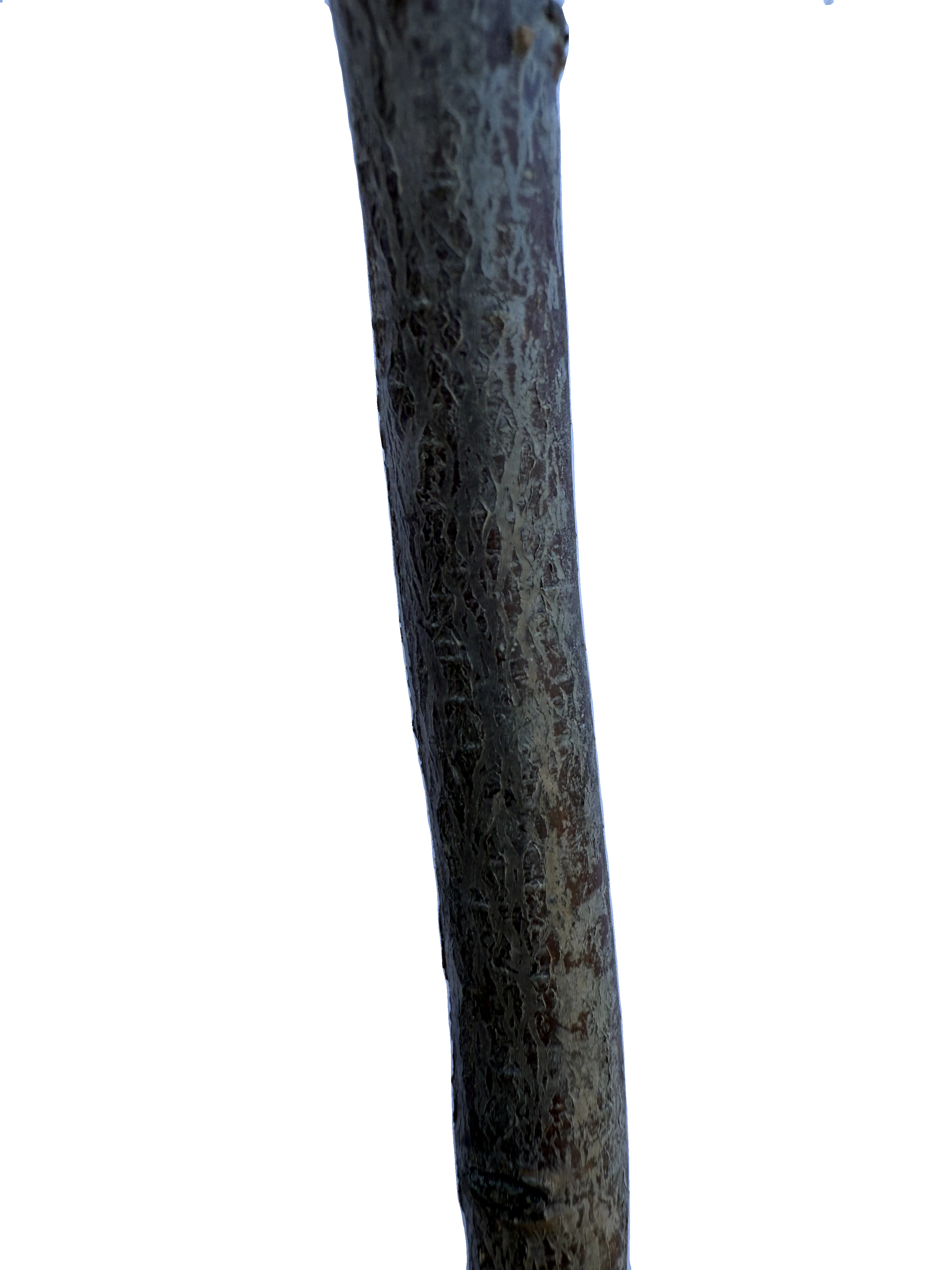

Supplement: Supplementary file 1 [file mmc1.zip › Angeleno Plum_RB.jpg]

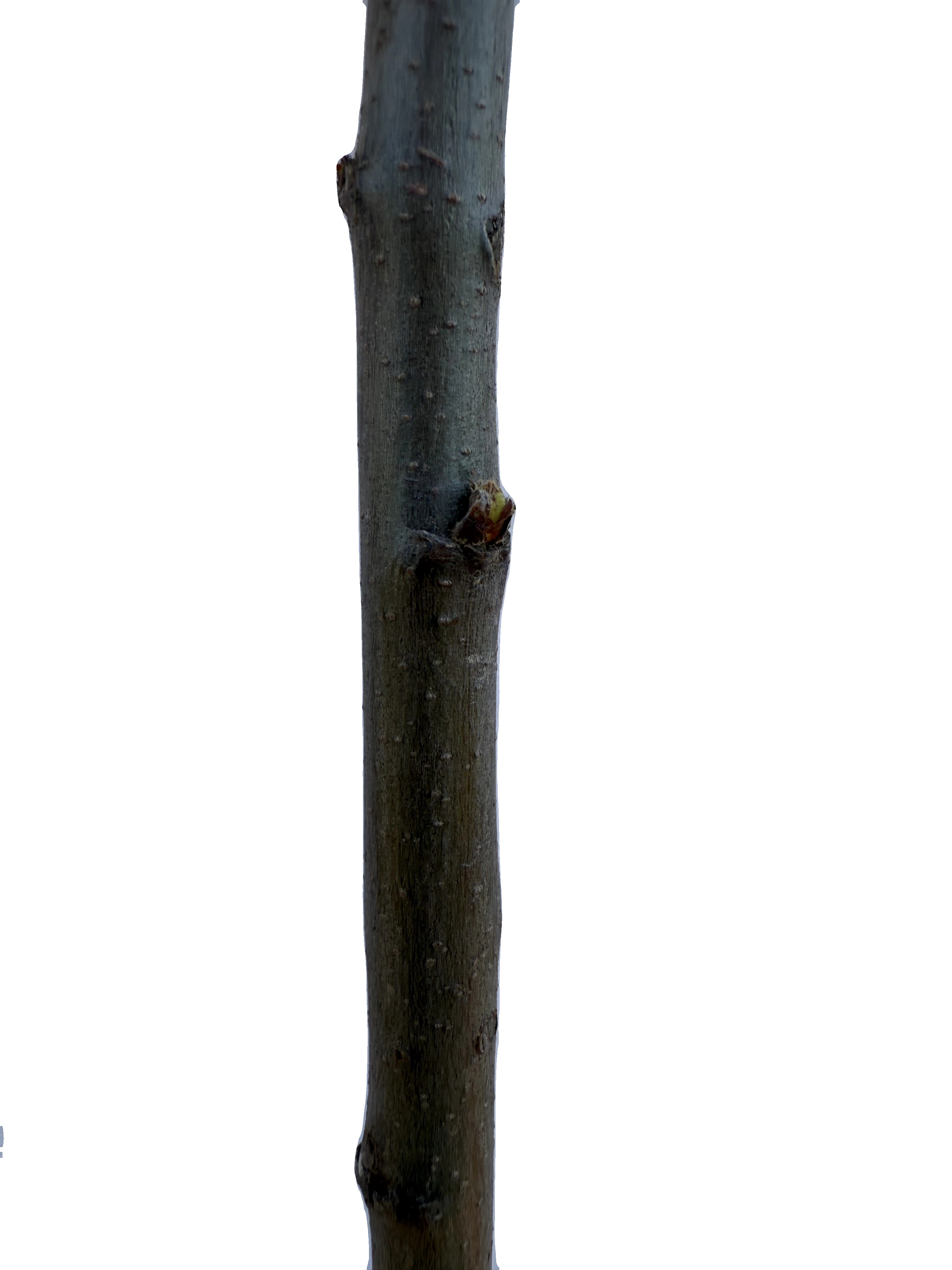

Supplement: Supplementary file 1 [file mmc1.zip › Ekmek Quince_RB.jpg]

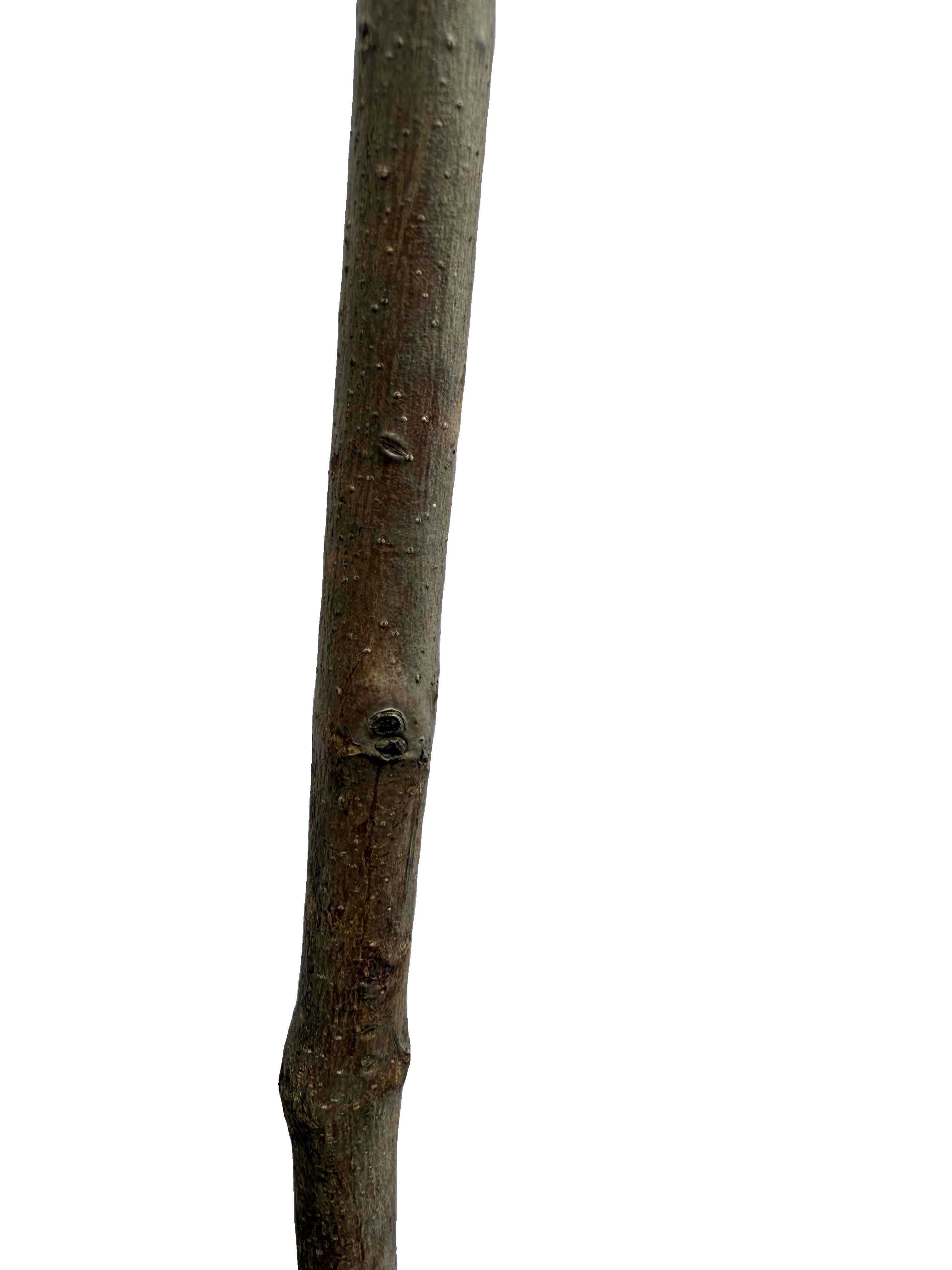

Supplement: Supplementary file 1 [file mmc1.zip › Jujube_RB.jpg]

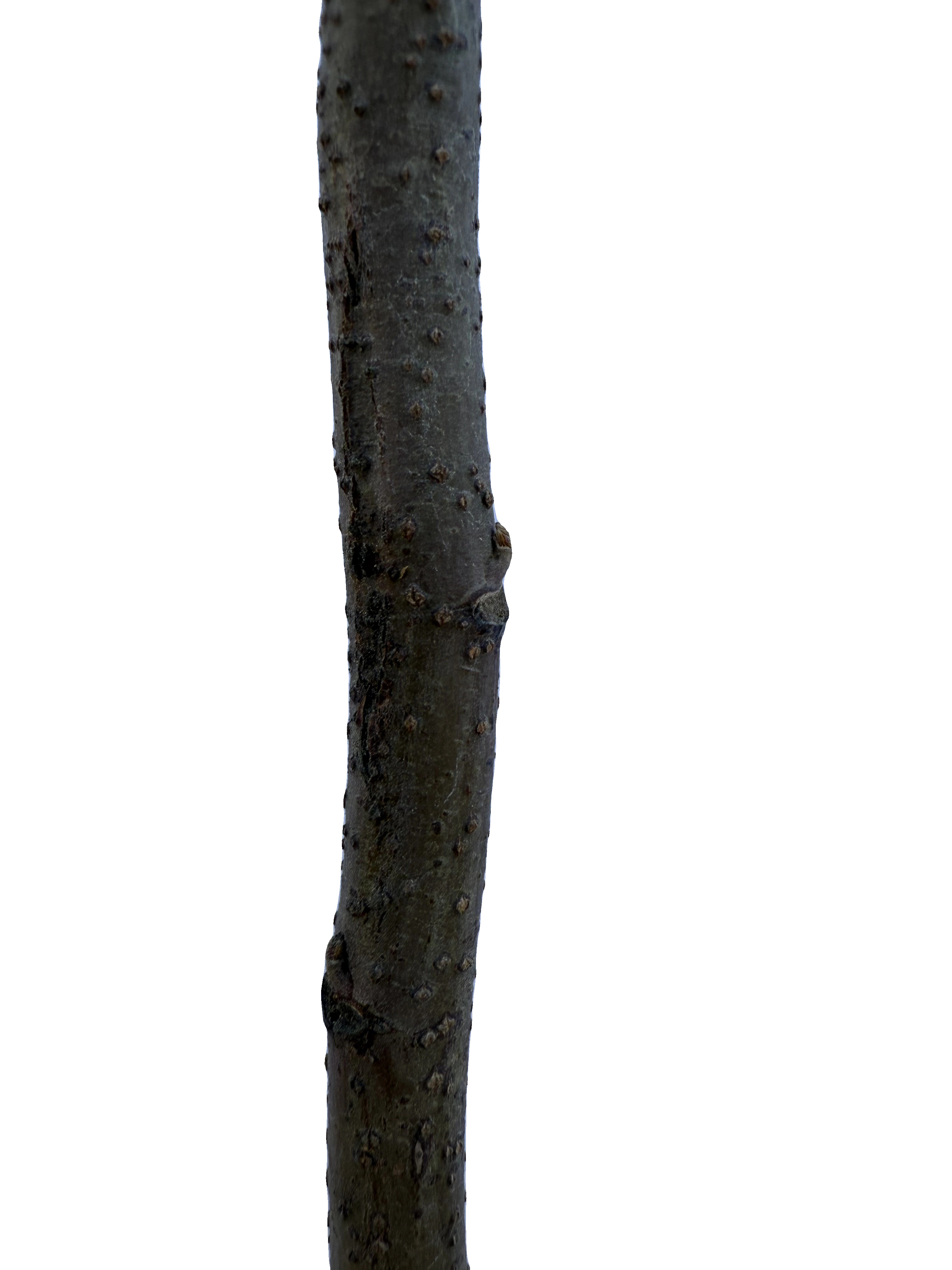

Supplement: Supplementary file 1 [file mmc1.zip › Carob_RB.jpg]
